# Alleviating *Clostridium perfringens*-Induced Intestinal Lesions in Chickens Using the Xylanase *Cb*Xyn10C and Its Binary Cocktail with a Protease

**DOI:** 10.3390/ani15020123

**Published:** 2025-01-07

**Authors:** Wenjing Zhang, Zhenzhen Hao, Daoxin Yang, Wangli Ji, Kairui Guo, Xianhua Sun, Shuai Wang, Shuyan Yang, Jianshuang Ma, Tong Wang, Huiying Luo, Bin Yao, Meiling Zhang, Yuan Wang, Huoqing Huang, Xiaoyun Su

**Affiliations:** 1 State Key Laboratory of Animal Nutrition and Feeding, Institute of Animal Sciences, Chinese Academy of Agricultural Sciences, Beijing 100193, China; 2 Laboratory of Aquaculture Nutrition and Environmental Health (LANEH), School of Life Sciences, East China Normal University, Shanghai 200241, China

**Keywords:** necrotic enteritis, *Clostridium perfringens*, xylanase, protease, broiler chickens, gut microbiota modulation

## Abstract

*Clostridium perfringens* infection can induce necrotic enteritis and lead to significant economic loss to the chicken industry. In this study, a xylanase (*Cb*Xyn10C), which effectively promotes the growth of probiotics, and a protease, which degrades the biofilm of *C. perfringens,* were analyzed for their ability to alleviate *C. perfringens*-induced necrotic enteritis in broiler chickens. A total of 300 male AA chickens were divided into five treatment groups (control, no enzyme and no *C. perfringens* challenge; Cp, no enzyme, *C. perfringens* challenge; Xyn, *Cb*Xyn10C plus *C. perfringens* challenge; Xyn+Am, *Cb*Xyn10C+Amylase plus *C. perfringens* challenge; Xyn+Ap, *Cb*Xyn10C+Alkaline protease plus *C. perfringens* challenge). The *C. perfringens* CVCC 60102 was administered orally on a daily basis to the chickens from 14 to 20 days. In comparison with Cp, Xyn+Ap significantly reduced intestinal damage in the duodenum, jejunum, and ileum of chickens challenged with *C. perfringens* (*p* < 0.05). The enzymes, and particularly Xyn+Ap, notably enhanced the expression of key intestinal barrier genes, reduced the IL-6 level, and decreased the DAO (diamine oxidase) level. Not unexpectedly, feeding enzymes influenced the abundance of *Lactobacillus* and *Butyricicoccus* bacteria in the intestine. These results indicated that *Cb*Xyn10C and protease can be used to alleviate intestinal damage caused by *C. perfringens* infection.

## Simple Summary

The *Clostridium perfringens* (*C. perfringens*)-induced necrotic enteritis (NE) is one of the major threats to the chicken industry, which is characterized by severe intestinal lesions. Although antibiotics can be used to control *C. perfringens* infection, there is worldwide concern about the possibility of developing antibiotics-resistant bacteria. In this study, based on the hypothesis that enzymes bearing the ability to affect growth of certain gut microbes could potentially alleviate the gut lesions incurred by *C. perfringens*, the performance of three xylanases was first compared, and *Cb*Xyn10C was found to be superior to the other two enzymes in stimulating more rapid growth of selected gut probiotics. An alkaline protease was further discovered to inhibit biofilm formation of *C. perfringens* in vitro. While feeding *Cb*Xyn10C to the broiler chickens alone could alleviate the gut lesions incurred by *C. perfringens*, supplementing with the protease, but not an amylase, further improved the resistance of the chickens to this bacterial challenge. Feeding the xylanase and its binary mixtures also tended to restore the concentrations of butyric acid and lactic acid and the gut microbiota to normalcy in chickens challenged with *C. perfringens*.

## 1. Introduction

Necrotic enteritis (NE) has been reported to cause an economic loss of as high as USD 6 billion per year [1]. In acute NE, the mortality rate can be up to 50% in broiler chickens; however, even subacute NE can cause intestinal lesions and reduce the performance of broiler chickens [2]. *Clostridium perfringens* (*C. perfringens*) is the causative pathogenic bacterium of NE, which propagates in the intestinal tract of chickens during the disease progression [3]. Based on its secreted toxins, *C. perfringens* can be classified into seven types (A, B, C, D, E, F, and G), with the types A and C most often found in NE in chickens [4]. Broiler chickens at 14–21 days of age are the most susceptible to this small intestine-damaging disease. In addition, environmental and nutritional stresses can promote the proliferation of *C. perfringens* in broiler chickens.

Although antibiotics are frequently used to control *C. perfringens* infection, the rapidly developing bacterial resistance to antibiotics raises widespread concerns around the world [5]. According to a recent review, 4.95 million people died due to bacterial antibiotic resistance in 2019 [6]. This suggests that other non-antibiotic maneuvers must be taken to deal with pathogenic bacteria infection (including *C. perfringens* infection), particularly those in the livestock and poultry industries. Currently, a number of agents, such as probiotics, prebiotics, and essential oils, have been explored as feed additives to combat *C. perfringens* infection. For instance, *Lactobacillus johnsonii*, *Clostridium butyricum*, fermented polysaccharides of *Hericium caputmedusae*, manno-oligosaccharides, and mannanase not only exhibit an antibacterial effect in vitro but also enhance intestinal integrity, reinforce the mucosal barrier, decrease the expression of anti-inflammatory factors, and mitigate oxidative damage in broiler chickens [6,7,8,9,10,11]. However, the preparation of live probiotic bacteria often requires strict storage conditions and is greatly influenced by environmental factors, thereby limiting its application potential. On the other hand, prebiotics and essential oils normally have a high cost upon usage, which is economically unaffordable in the poultry industry.

Many of these antibiotics-replacing agents assist in combating *C. perfringens* infection essentially by exerting a role in modulating the gut microbiota. Enzymes are specialized biocatalysts that can also have a significant impact on changing the composition of a gut microbiome [12]. Unlike probiotics and prebiotics, an additional advantage of enzymes is that highly efficient and stress tolerant enzymes can be produced cheaply in large quantities [13]. Based on these facts, it is reasonable to hypothesize that using enzymes to manipulate the gut microbiota could be a potentially effective and economically viable strategy to control NE in broiler chickens [14]. Indeed, there have been several reports documenting that those enzymes (e.g., a lytic enzyme and *β*-mannanase) can be used to control NE [15,16].

Among the many enzymes added to feeds, xylanase has long been utilized to depolymerize xylan in the plant cell wall of feeds, which is also known as one of the anti-nutrient factors [11]. The most extensively studied xylanases are the GH10 and GH11 families, which differ from each other in both amino acid sequence and structure. In comparison to the GH11 xylanases, the GH10 counterparts are more diversified and appear to produce relatively simpler xylo-oligosaccharides products [17]. Xylanases reduce the viscosity of chyme and facilitate nutrient release in broiler chickens. By shortening the retention time of chyme in the digestive tract, the use of xylanases in the feeds could lead to an additional benefit by significantly reducing the colonization rate of pathogenic bacteria [18].

In terms of using xylanases as an enzyme to degrade the anti-nutrient factor xylan, although some xylanases are reported to enhance animal growth performance and immune function [19], there are also conflicting results from the others [18,20]. It is worth noting that most, if not all, studies primarily considered using xylanases to facilitate nutrient release from feeds and focused on the impact of the xylanases on growth performance. Nonetheless, xylanase has an additional trait that has not been well exploited. These glycoside hydrolases can release different xylo-oligosaccharides from xylan, thereby promoting growth of certain gut probiotic bacteria and, particularly, the lactobacilli and bifidobacteria probiotics [21,22]. Note that the gut is a highly crowded environment, with commensals, probiotics, and pathogens possibly competing for the same carbon and energy supplies [23,24]. Such interaction of gut bacteria renders probiotics a powerful tool for modulating the gut microbiota and improving the host health [25]. For example, *Clostridium butyricum* HJCB998 could reduce the abundance of *Escherichia coli*, *Salmonella*, and *C. perfringen* and promote growth of *Lactobacillus* and *Bifidobacterium* in the cecum of broiler chickens [26].Therefore, it is reasonable to hypothesize that a deliberately selected xylanase could potentiate growth competence of some probiotics, which then outcompete and mitigate the infection of *C. perfringens*.

Pathogenic bacteria have the ability to produce biofilms, which are dynamic bacterial extracellular aggregates characterized by diverse composition [27]. The biofilms serve as a barrier, assisting the pathogens in defending against unfavorable environmental changes [28]. Nature has evolved enzymes possessing the ability to degrade the biofilm’s components, thereby disrupting the integrity of the biofilm structures and rendering the pathogens more susceptible to attack [29]. Supplementation with such a biofilm-degrading enzyme to the selected xylanase might further reduce the growth of *C. perfringens* infection and help to alleviate the NE symptoms in chickens.

## 2. Materials and Methods

### 2.1. Feed Enzymes

The *Cb*Xyn10C xylanase from *Caldicelluliruptor bescii* used in this study was recombinantly expressed in the yeast *Pichia pastoris* (*P. pastoris*) GS115-pPIC9*γ* in our laboratory as described previously [30]. *Np*XynC (*p*) from *Neocallimastix patriciarum* was recombinantly expressed in *P. pastoris* [31]. *Np*XynC (*t*) was recombinantly expressed in *Trichoderma reesei* and, therefore, had additional xylanase activity from this filamentous fungus. The alkaline protease (Ap) from *Parengyodontium album* was also expressed in *P. pastoris* [32]. The mesophilic α-amylase (Amy) was obtained from *Bacillus amyloliquefaciens*. *C. perfringens* CVCC 60102 was a gift from Prof. Kui Zhu of China Agricultural University.

### 2.2. Using Xylanase-Degraded Wheat-Soybean to Support Growth of Probiotic Bacteria

The probiotic strains used in this study were maintained in our lab, with the detailed method of isolation given below. The 12 probiotics were *Lactobacillus reuteri*, *Lactobacillus ingluviei*, *Lactobacillus johnsonii*, *Lactobacillus crispatus*, *Lactobacillus mucosa*, *Lactobacillus salivarius*, *Lactobacillus brevis*, *Enterococcus hirae*, *Enterococcus faecium*, *Enterococcus durans*, *Pediococcus pentosaceus*, and *Pediococcus acidilactici*. To isolate the strains, 1 g of the feces or intestinal contents of broiler chickens was transferred to 100 mL sterile water, mixed, and left standing for 10 min. One mL of the supernatant was added to the MRS medium (i.e., de Man, Rogosa and Sharpe medium, abbreviated as MRS) and then cultured anaerobically at 37 °C for 2 d. The culture was diluted to 10^−1^ to 10^−6^ and 100 μL each of the diluted cultures were spread on MRS agar plates and incubated anaerobically at 37 °C for 3 d. Bacterial colonies were successively streaked on MRS agar plates for purification. The 16S rRNA of each isolated bacterial single colony was sequenced.

A wheat-soybean mixture (0.53 g wheat + 0.27 g soybean) was degraded using three xylanases (*Np*XynC (*p*), NpXynC (*t*), *Cb*Xyn10C) in a reaction system consisting of 0.8 g substrate and 200 U xylanase. The reaction buffer used was Tris-HCl at pH 7.4, and the total volume of the reaction mixture was 40 mL. The reaction was carried out at 50 °C for 24 h with continuous rotation. The resulting supernatant, obtained after degradation, was used as a carbon source to support the growth of the above-mentioned probiotic bacteria. The probiotics were then recovered, washed with sterile water, and resuspended in glucose-free MRS medium to achieve an OD_600_ of approximately 1.7. The final tubes contained a medium consisting of 3 mL glucose-free MRS (at a 4/3-fold concentration) without glucose and 1 mL of volume of the collected supernatant. The control tubes contained glucose-free MRS medium and a wheat-soybean mixture soaking solution obtained under the same conditions. Each tube was inoculated with different probiotics at a 3% (*v*/*v*) inoculum. Three replicates were prepared for each tube, and the OD_600_ was measured at regular intervals.

The xylanases in the degradation products of the wheat-soybean mixture were inactivated by boiling. Then the degradation product was appropriately diluted and 10 μL of the sample was taken out for analysis using high-performance anion-exchange chromatography pulsed amperometric detector assay (HPAEC-PAD) for 40 min. The detection conditions were as follows: Dionex CarboPac™ PA-100 column (8.5 μm, 4 × 50 mm, Thermo, Bremen, Germany); mobile phase, 1 M NaOH; flow rate, 0.55 mL/min.

### 2.3. Inhibiting Growth of C. perfringens by the Probiotic Bacteria

The effect of the 12 probiotic bacteria in inhibiting *C. perfringens* was assessed using the agar diffusion method. The probiotics were individually cultured in the MRS medium and *C. perfringens* was cultured in the fluid thioglycollate (FTG) medium. Both kinds of bacteria were grown at 37 °C under anaerobic conditions overnight until their OD_600_ reached 1.2. Then, the tryptose-sulfite-cycloserine (TSC) agar medium incubated at 37 °C was mixed with the *C. perfringens* culture (*v*/*v*: 40:1). Five mL of this mixture was then dispensed into each well of the 6-well cell petri dishes. Ten μL of the probiotic turbid liquid was spotted onto the center of each well after the medium solidified. The petri dishes were cultured at 37 °C under anaerobic conditions for 12 h and the radii of the antibacterial zones were individually measured.

### 2.4. Biofilm Degradation

*C. perfringens*, in the fluid thioglycollate without agar medium (FT), was cultured anaerobically at 37 °C for 12 h to reach an OD_600_ of 0.8. The culture was then dispensed into 24-well plates at a volume of 500 μL per well. The plates were further anaerobically cultured at 37 °C for 3 d. Afterwards, xylanase, alkaline protease (both at final concentrations of 1.0, 1.5, 2.0, 2.5, 3.0, and 3.5 U/mL, respectively), and mesophilic *α*-amylase (at final concentrations of 0.1, 0.25, 0.5, 0.75, and 1 U/mL, respectively) were added to each well. The control group was added with an equal volume of phosphate buffer (0.1 M, pH 5.5). The incubation was continued for 6 h. Finally, the biofilm degradation was assessed using the crystal violet staining method with slight modifications [33]. Briefly, the wells were gently washed with 0.9% NaCl twice, fixed with methanol for 10 min, thoroughly dried, and stained with 0.5% crystal violet for 15 min. After staining, the wells were rinsed gently with water twice, dried, and 0.3 mL of 30% acetic acid solution was added to dissolve the crystal violet. The optical density at 570 nm (OD_570_) was measured. The degradation rates of biofilm were calculated using the following formula: (OD_570_PBS_ − OD_570___enzyme_)/OD_570___PBS_ × 100%.

### 2.5. Animal Experimental Design and Management

The Experimental Animal Welfare and Ethical of Institute of Animal Science, Chinese Academy of Agriculture Sciences approved all the procedures involved in this experiment (Approval number: IAS2022-106).

A total of 300 healthy, one-day-old male white feather broilers were randomly divided into the 5 treatments, with 6 replicates per treatment and 10 birds per replicate. Wheat contains higher amounts of xylan (8.02%) than corn (6.19%) and thus may serve as a better substrate for xylanase [11]. Therefore, the basal diet used was powdered wheat-soybean meal instead of corn-soybean meal (Table 1). Two control groups were designed, i.e., the control and Cp groups receiving the basal diet without or with *C. perfringens* challenge, respectively. The experimental treatment groups included the enzymes *Cb*Xyn10C (Xyn), *Cb*Xyn10C+Amylase (Xyn+Am), and *Cb*Xyn10C+Alkaline protease (Xyn+Ap), respectively, all with *C. perfringens* challenge. The enzymatic activities of *Cb*Xyn10C, Amylase, and Alkaline protease in the prepared feed were 2500 U/kg, 500 U/kg, and 2500 U/kg, respectively. These were consistent with an in vitro bionic experiment, which indicated that these concentrations could allow for the release of more nutrients from the feeds. The broilers were orally administered 1 mL of *C. perfringens* (containing 3 × 10^8^ CFU) daily for 7 days (14 d–20 d), while the broilers in the control group were fed an equal volume of the culture medium. All birds were slaughtered for sampling at 21 d. The body weight (BW) of the broilers was recorded on day 13 and day 20. Meanwhile, the feed weight was measured, allowing for the calculation of average daily gain (ADG), average daily feed intake (ADFI), and feed conversion ratio (FCR).

The study was conducted in the China Agricultural University Zhuozhou Experimental Station. The feeding environment and management conditions of the broilers in each pen were consistent and met the feeding requirements. Feeding management followed the routine management and immunization procedures of the experimental station. Broilers had free access to feed and water throughout this experiment. For the first 3 d, there was 24 h of light, which was gradually reduced to 16 h to enhance the broilers’ anti-stress ability.

### 2.6. Sample Collection

On day 21, one chicken with a body weight similar to the average in each pen was selected and killed by intracardial administration of sodium pentobarbital (30 mg/kg of BW) followed by jugular exsanguination. The stool score was based on the feces status of the broiler chickens and the cleanliness around the cloaca [34]. The standards were as follows: 0, normal feces; 1, soft stools; 2, diarrhea; 3, stuck to the cloaca.

The vessels with collected blood were left for 30 min before being centrifuged at 3500 rpm for 10 min and 4 °C. The serum was separated and stored at −20 °C. Each sampled intestinal segment (duodenum, jejunum, and ileum) was cut in the middle, with the chyme rinsed out using normal saline. Two cm each of the intestinal segment were taken from the middle of duodenum, jejunum, and ileum. The luminal chyme was gently flushed with saline. One cm of the intestinal segment was processed using autoclaved surgical scissors to form 0.2 cm loops. These loops in the centrifuge tubes were then promptly placed into liquid nitrogen for quick freezing. The remaining 1 cm of the intestinal segment was fixed with 4% paraformaldehyde. The other segments of the small intestine were dissected longitudinally to remove chyme and evaluated for intestinal damage. The cecal digesta was collected in sterilized centrifuge tubes and rapidly frozen using liquid nitrogen.

### 2.7. Serum Indexes

The levels of immunoglobulin G (IGG) and immunoglobulin M (IGM) in the serum were determined using a Chicken Immunoglobulin G ELISA Kit (catalog number: CH50007) and a Chicken Immunoglobulin M ELISA Kit (catalog number: CH50015) from Beinglay (Wuhan, Hubei Province, China). The levels of immunoglobulin A (IGA), diamine oxidase (DAO), interleukin 1*β* (IL-1*β*), interleukin 6 (IL-6), interleukin 10 (IL-10), and tumor necrosis factor-*α* (TNF-*α*) were measured using the ELISA Kits specific to each by Colorful-Gene Biotech in Wuhan (Chicken sIgA ELISA Kit, catalog number: JYM0036ch; Chicken DAO ELISA Kit, catalog number: JYM0113ch; Chicken IL-1*β* ELISA Kit, catalog number: JYM0041ch; Chicken IL-6 ELISA Kit, catalog number: JYM0028ch; Chicken IL-10 ELISA Kit, catalog number: JYM0040Ch; and Chicken TNF-*α* ELISA Kit, catalog number: JYM0033ch).

### 2.8. Intestinal Lesion Score

The duodenum, jejunum, and ileum were all used to evaluate the extent of the lesions. A lesion score was determined using the following criteria: 0, no obvious injury; 1: 1–5 hemorrhagic lesions; 2: 6–10 hemorrhagic lesions; 3: 11–15 hemorrhagic lesions; 4: >15 hemorrhagic lesions; 5: flaky hemorrhagic, necrosis or ulceration.

### 2.9. Intestinal Morphology

The fixed duodenum, jejunum, and ileum samples were embedded in paraffin wax, stained with hematoxylin and eosin (HE), and sectionalized. The stained sections were scanned panoramically using the Digital Slice Scanning System. The villi height (VH) and crypt depth (CD) were measured at 40× magnification and the ratio of the two was calculated (VH/CD) in four villi from each slice.

### 2.10. Expression of Key Genes Maintaining the Intestinal Barrier

The total RNA of duodenum, jejunum, and ileum tissues was isolated using a Trizol reagent. The cDNA was synthesized using a HiScript^®^ 1st Strand cDNA kit following the manufacturer’s protocol (Vazyme Biotech, Nanjing, China). The primers used for quantitative real-time PCR analysis of chicken intestinal barrier cytokines and *β*-actin gene are listed in Table S1. The QuantStudio 6 Flex and 2 × ChamQ Universal SYBR qPCR Master Mix from Vazyme Biotech were used to determine the expression level of selected genes.

Equivalent amounts of cDNA from each sample (200 ng) were used in the amplification. The PCR reactions were carried out in the following manner: 95 °C for 3 min, then 40 cycles of 95 °C × 30 s, 95 °C × 10 s, and 60 °C × 30 s. A 2^−∆∆Ct^ method was used to compare the expression level of selected genes [14].

### 2.11. Determining the Butyric Acid and Lactic Acid in the Cecal Content

The cecal content was freeze-dried. A portion of ~50 mg was taken and soaked with 1 mL deionized water at 4 °C for 12 h. Subsequently, the sample was centrifuged at 12,000 rpm and 4 °C for 15 min. The supernatant was mixed with 25% metaphosphate at a 5:1 volume ratio and kept at −80 °C overnight. Then, the mixture was thawed and centrifuged again at 12,000 rpm and 4 °C for 15 min. The supernatant was filtered through 0.22 μm membrane filters and used for SCFA (short-chain fatty acids) and lactic acid detection. The high-performance liquid chromatography (HPLC, SPD20A, Shimadzu, Tokyo, Japan) equipped with an Agilent ZORBAX SB-C18 column (5 µm, 4.6 × 250 mm, Santa Clara, CA, USA) was used to detect butyric acid and lactic acid. They were detected at 213 nm for 30 min. A total of 10 μL each of the samples was used for HPLC, and the HPLC conditions were as follows: mobile phase, methanol/0.01 M NaH_2_PO_4_ (28:72, *v*/*v*, adjusting the pH to 2.45 with phosphoric acid); flow rate, 0.75 mL/min; column temperature, 30 °C.

### 2.12. Gut Microbiota Analysis

The cecum chyme was sent to Shanghai Meiji Biomedical Technology Co., Ltd. (Shanghai, China) for determination of the microbial diversity. The bacterial 16S rRNA gene was used to determine the intestinal flora diversity. Normalized concentrations of purified genomic DNA were used as templates for analysis of the microbial community for Illumina sequencing. The V3-V4 regions of 16S rDNA in the intestinal flora were amplified with the primers 338F (5′-ACTCCTACGGGAGGCAGCA-3′) and 806R (5′-GGACTACHVGGGTWTCTAAT-3′) [14]. The paired-end (PE) reads obtained from Illumina sequencing were first spliced based on their overlap relationship. The sequence quality was also controlled and filtered, and sequences with a 97% sequence similarity level were selected. The selected sequence reads were then clustered into operational classification units (OTUs) from scratch. The data were further analyzed via OTU clustering analysis and species taxonomic analysis. Additionally, tests of the sequencing credibility were performed. Clustering was carried out based on the taxonomic information at both the phylum and genus levels, and the community structure was statistically analyzed. The abundance of microbial taxa was expressed as a percentage of the total 16S rRNA gene sequences, and differences between treatments were compared using the Kruskal–Wallis H test.

### 2.13. Statistical Analysis

The results were displayed as mean ± SEM (Standard Error of the Mean). All parameters for each bird from each treatment group were analyzed across all treatment groups via one-way ANOVA analysis using the IBM SPSS Statistics 20.0, except for the cecal microbial data, intestinal lesion scores, and stool scores. When a significant difference was observed (*p* < 0.05), the Student–Newman–Keuls test was used for pairwise comparison. The cecal microbial data, intestinal lesion scores, and stool scores were analyzed via a non-parametric Kruskal–Wallis test. Differences at *p* < 0.05 were considered significant.

## 3. Results

### 3.1. CbXyn10C Stimulated Rapid Growth of Probiotic Bacteria

**Figure 1:**
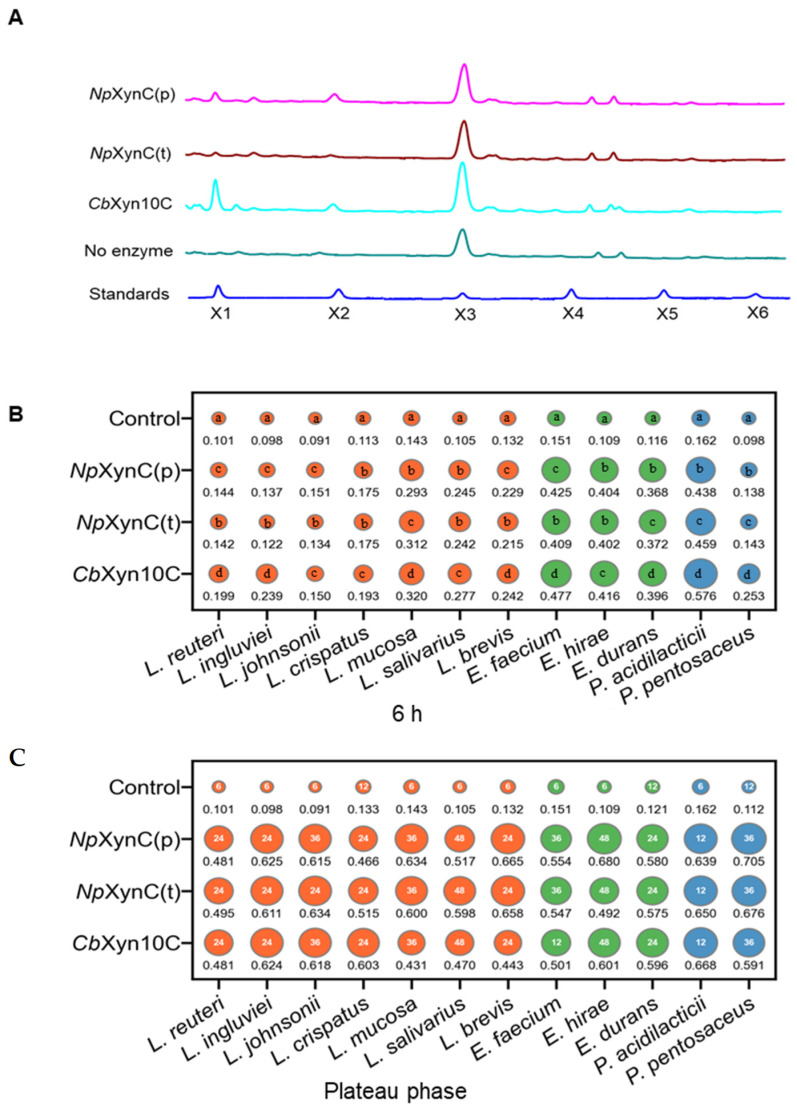
Effect of three xylanases on growth of selected gut bacteria. (**A**) The degradation products released from the wheat/soybean mix by the three xylanases as analyzed via HPAEC-PAD. (**B**) The growth of thirteen gut bacteria stimulated by xylo-oligosaccharides as determined by the optical density at 600 nm (OD_600_) at 6 h (**B**) and the plateau phase (**C**). The numbers under the dots are the OD_600_ (the average of three biological replicates) and the sizes of the dots are proportional to the OD_600_. The numbers in the dots are the time (h) needed for the maximal growth of each strain. Different letters in two circles for the same bacterium indicate statistical difference (*p* < 0.05). X1, xylose; X2, xylobiose; X3: xylotriose; X4: xylotetraose; X5: xylopentaose; X6: xylohexaose.

To understand how xylanases could play different roles in stimulating bacterial growth, three xylanases—i.e., *Cb*Xyn10C from *C. bescii* (recombinantly expressed in *P. pastoris*), *Np*XynC (*p*) from *N. patriciarum* (recombinantly expressed in *P. pastoris*), and *Np*XynC (*t*) (recombinantly expressed in *T. reesei*, bearing additional cellulase activity)—were selected for investigation. The same amounts of the three xylanases (250,000 U per kg of feeds) were used to degrade a wheat/soybean mixture. For *Cb*Xyn10C, the degradation products were determined to contain mainly xylotriose and minor amounts of xylose and xylobiose (Figure 1A). Note that, although the wheat/soybean mixture had a peak of xylotriose, in all enzyme treatments, the xylotriose peaks were higher than those in the no enzyme control group. For *Np*XynC (*p*), the released xylose was less than that from *Cb*Xyn10C. For *Np*XynC (*t*), the released amount of xylose further decreased in comparison with those from *Cb*Xyn10C and *Np*XynC (*p*). These xylooligosaccharides were incubated with twelve gut bacterial isolates, including *L. reuteri*, *L. ingluviei*, *L. johnsonii*, *L. crispatus*, *L. mucosa*, *L. salivarius*, *L. brevis*, *E. faecium*, *E. hirae*, *E. durans*, *P. acidilacticii*, and *P. pentosaceus*. It was discovered that all xylanases could stimulate the selected gut bacteria to their maximal growth at 36 h (Figure 1B). However, some bacteria differed in their initial growth rates. For such bacteria (*L. reuteri*, *L. ingluviei*, *L. mucosa*, *L. brevis*, *E. durrans*, *P. acidilacticii*, and *P. pentosaceus*), *Cb*Xyn10C clearly stimulated growth faster than *Np*XynC (*p*) and *Np*XynC (*t*) at 6 h. This rapid propagation could help bacteria to establish growth competence in the highly crowded gut. Therefore, *Cb*Xyn10C was selected for further study.

### 3.2. Inhibiting Growth of C. perfringens by Probiotic Bacteria

The inhibitory effect of the twelve gut bacterial isolates on the growth of *C. perfringens* was determined using the agar diffusion method. Upon incubation, large halos appeared for *L. reuteri*, *L. ingluviei*, *L. mucosa*, *L. salivarius*, *P. acidilacticii*, *P. pentosaceus*, and *E. hirae*, with a zone diameter > 1 cm. Smaller halos with diameters of 0.5–1 cm were observed for *E. faecium* and *E. durans* (Table S2). This indicated that all of the selected gut bacteria could inhibit *C. perfringens* propagation.

### 3.3. Inhibiting C. perfringens Biofilm Formation by the Protease

**Figure 2:**
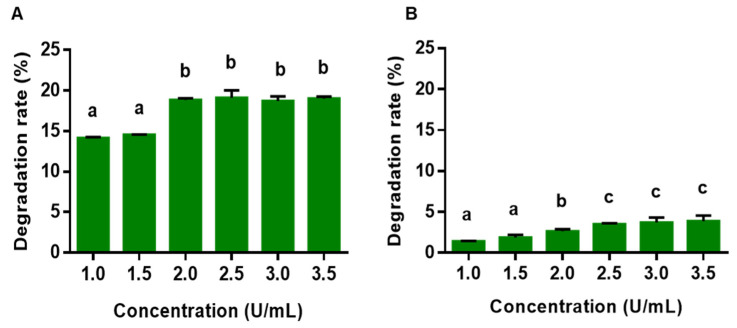
Destruction of the biofilm of *C. perfringens* by the enzymes: (**A**) protease; (**B**) *Cb*Xyn10C. The bacterium *C. perfringens* was cultured in 24-well plates to allow for the formation of biofilms. Then, the enzymes (*Cb*Xyn10C and protease) were added at 37 °C and the incubation was continued for 6 h. The amounts of biofilms were determined using the crystal violet method. The data are shown as mean ± SEM (*n* = 6) and the different letters on the two columns indicate statistical difference (*p* < 0.05).

It was hypothesized that incubating *C. perfringens* with a protease could impair the formation of its biofilms. Indeed, treating *C. perfringens* with gradually increasing concentrations (1 to 3.5 U/mL) of an alkaline protease from *P. album* could decrease the formation of biofilm from 14.1% to 19.0% (Figure 2A). In contrast, the xylanase *Cb*Xyn10C degraded the biofilm to a much smaller extent (Figure 2B). An amylase control from *Bacillus amyloliquefaciens* had nearly no effect on biofilm degradation.

### 3.4. Effect of CbXyn10C and Its Binary Cocktail on Growth Performance

The *Cb*Xyn10C xylanase was used either alone (Xyn group) or in combination with the amylase (Xyn+Am group) or alkaline protease (Xyn+Ap group) to feed broiler chickens from day 1 to 20. The chickens were subjected to *C. perfringens* challenge from day 14 to 20. Firstly, the effect of *Cb*Xyn10C and its binary cocktail on the growth performance of chickens in this *C. perfringens-*challenged period was determined. However, there appeared to be no significant differences in ADG, ADFI, and FCR from day 1 to 20 in all the groups (Table 2). During the challenge, the ADG and ADFI of the Cp group were significantly lower than those of the control group (*p* < 0.05). Compared to the Cp group, the addition of Xyn+Am significantly improved ADG (*p* < 0.05). Furthermore, the BW at 20 days in the Xyn+Am and Xyn+Ap group were significantly greater compared to both the Cp and Xyn group (*p* < 0.05). The BW of broiler chickens with Xyn+Ap at 14 days was significantly higher than those of the other groups (*p* < 0.05).

### 3.5. CbXyn10C and Its Binary Mixture with the Protease Reduced the Intestinal Lesion in Chickens Challenged with C. perfringens

**Figure 3:**
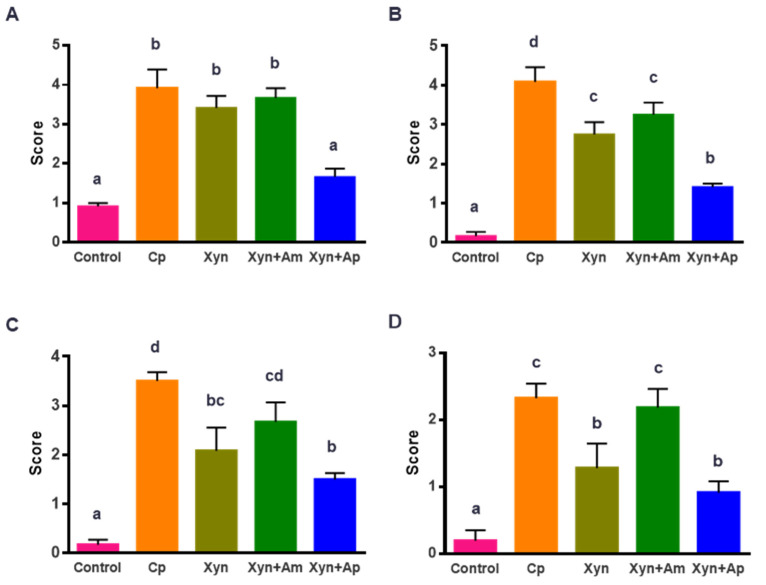
Effect of supplementing with the xylanase *Cb*Xyn10C and its binary combination with one of the two other enzymes on the gut health in broiler chickens challenged with *C. perfringens*. (**A**–**C**) The intestinal injury scores for the duodenum (**A**), jejunum (**B**), and ileum (**C**) in broilers challenged with *C. perfringens* and fed with one of the enzymes. The scoring standards were set as follows: 0, no obvious injury; 1: 1–5 hemorrhagic lesions; 2: 6–10 hemorrhagic lesions; 3: 11–15 hemorrhagic lesions; 4: >15 hemorrhagic lesions; 5: flaky hemorrhagic, necrosis or ulceration. (**D**) The stool scores in broilers challenged with *C. perfringens* and fed with one of the enzymes. The standards were as follows: 0, normal feces; 1, soft stools; 2, diarrhea; 3, stuck to the cloaca. The data are shown as mean ± SEM (*n* = 6) and different letters on two columns indicate statistical difference (*p* < 0.05).

Lesions in the three segments of the small intestine of broilers were assessed, the statistical results for which are shown in Figure 3. The infection of *C. perfringens* in the animals significantly increased the intestinal lesion score and caused perceivable lesions in the duodenum, jejunum, and ileum (Figure 3A–C, *p* < 0.01). Compared to the Cp group (chickens challenged with *C. perfringens*), the Xyn group significantly reduced the lesion scores in the jejunum from 4.1 to 2.8 (Figure 3B) and in the ileum from 3.5 to 2.1 (Figure 3C). On the other hand, the Xyn+Am group had comparable lesion scores to those of the Xyn group for the jejunum and ileum. Not unexpectedly, the Xyn+Ap group had the least severe broiler intestinal lesions among the three enzyme cocktail treatment groups. The scores were decreased to only 1.7 (Figure 3A), 1.4 (Figure 3B), and 1.5 (Figure 3C) for the duodenum, jejunum, and ileum, respectively, which were all significantly lower than those in the Cp group. The data indicated that *Cb*Xyn10C and its combination with the protease could indeed alleviate the injury incurred by *C. perfringens* infection. In accordance with the lesion scores, supplementing the feeds with *Cb*Xyn10C and protease alleviated the diarrhea symptom, as reflected by the decreased score in the stool. Challenging with *C. perfringens* increased the stool score from 0.2 to 2.3, while adding *Cb*Xyn10C and *Cb*Xyn10C+Alkaline protease decreased the scores to 1.3 and 0.9, respectively (Figure 3D).

### 3.6. CbXyn10C and Its Enzyme Mixture with the Protease Helped to Maintain the Integrity of the Intestinal Barrier

Next, the morphology of the intestine in chickens was monitored by measuring the VH and CD using the paraffin slides stained with HE. The ratio of VH/CD was also calculated to evaluate the impact of the enzymes on the morphology of the small intestine and the development of intestinal cells (Table S3). The enzyme cocktail appeared not to have a significant effect on these values.

When comparing the expression levels of tight junction proteins and mucin, compared to the control group, the transcript abundance of the intestinal barrier genes—i.e., ZO-1, occludin, and MUC-2 in jejunum, claudin-1 and MUC-2 in ileum—was significantly decreased in chickens challenged with *C. perfringens* (*p* < 0.05, Table 3). Supplementation with Xyn led to a significant increase of occludin in the duodenum, claudin-1 in the jejunum, and ZO-1 in the ileum when comparing the values in the Cp group (*p* < 0.05, Table 3). Xyn+Am also significantly increased the expression of claudin-1 both in the duodenum and the jejunum, as well as occludin in duodenum and ZO-1 in the ileum, in comparison with the Cp group (*p* < 0.05, Table 3). Xyn+Ap significantly promoted the expression of three intestinal barrier marker genes (ZO-1, claudin-1, and occludin) in the duodenum, claudin-1 in the jejunum, and three genes (ZO-1, claudin-1, and occludin) in the ileum (*p* < 0.05, Table 3) in comparison with the Cp group. Collectively, the enzyme treatments could promote the expression of intestinal barrier factors.

### 3.7. Effect of the Enzymes on the Serum Biochemical Indicators

Compared to the control group, challenging the chickens with *C. perfringens* induced a highly significant increase in the serum diamine oxidase (DAO), a marker positively related to gut permeability (*p* = 0.007). Although there was no statistical difference, the addition of the enzymes Xyn+Ap tended to reduce this value (*p* = 0.067). There was no significant difference when comparing the immunoglobulins (IgA, IgG, and IgM). Although IL-6 and IL-1*β* were significantly increased after the challenge (*p* ≤ 0.003), adding Xyn, Xyn+Am, and Xyn+Ap significantly reduced IL-6 (*p* < 0.05). The serum level of TNF-*α* in the Cp group did not change when compared to that of control group. However, when the *Cb*Xyn10C and *Cb*Xyn10C+protease enzymes were used, the serum levels arose to significantly higher levels (Table 4).

### 3.8. Effect of Feeding Enzymes on Butyric Acid and Lactic Acid in the Cecal Chyme

**Figure 4:**
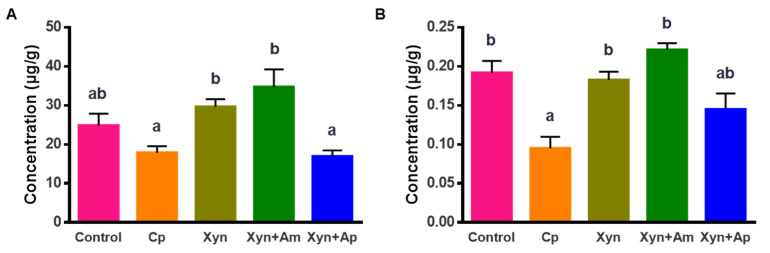
The lactic acid and butyric acid concentrations in the cecal content as determined using HPLC analysis: (**A**) butyric acid; (**B**) lactic acid. The data are shown as mean ± SEM (*n* = 6) and different letters on two columns indicate statistical difference (*p* < 0.05).

*C. perfringens* challenge induced a significant decrease in the concentrations of butyric acid (by 28.2%, Figure 4A) and lactic acid (50.4%, Figure 4B) in the cecal chyme when compared with those of the control group. Being fed Xyn and Xyn+Am restored broilers’ concentrations to normalcy at levels comparable to those of the control group. Supplementing with the protease decreased the concentration of lactic acid when compared with that of Xyn.

### 3.9. CbXyn10C and the Enzyme Cocktails Changed the Gut Microbiota

**Figure 5:**
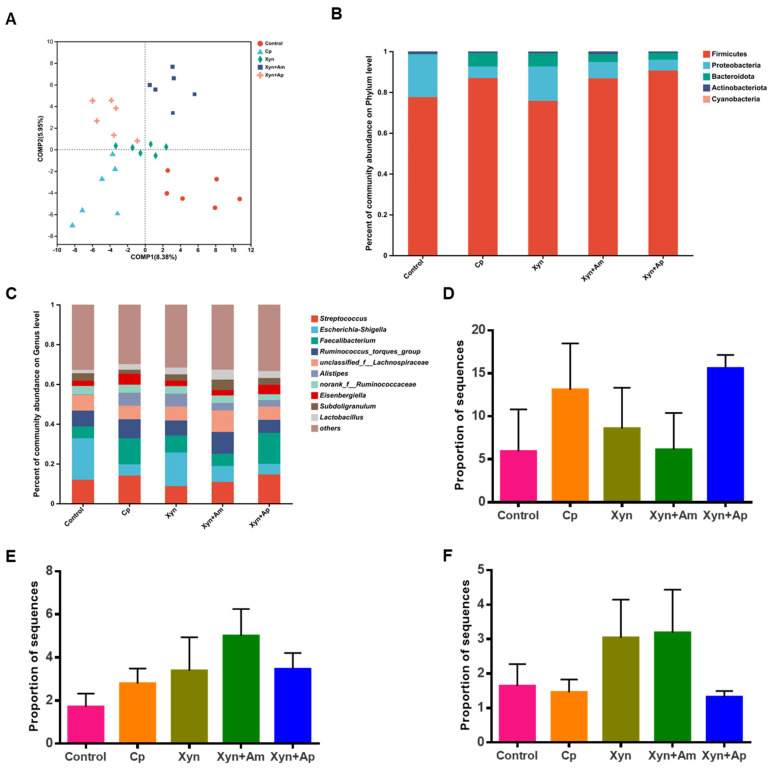
Feeding with enzymes changed the gut microbiota of the broiler chickens. (**A**) Partial least squares discriminant analysis (PLS-DA) of microbiota community at the genus level. (**B**,**C**) Relative abundance of the dominant bacterial communities at the phylum (**B**) and genus (**C**) level sampled from the cecal digesta. (**D**–**F**) The Kruskal–Wallis H test at the genus level for *Faecalibacterium* (**D**), *Lactobacillus* (**E**) and *Butyricicoccus* (**F**).

The PLS-DA analysis showed a clear distribution of the gut microbiome samples in four quadrants, indicating that both the *C. perfringens* challenge and the enzyme feeding influenced the composition of the gut microbiota (Figure 5A). The sample richness and diversity are presented in Table S4. The Good’s coverage was nearly 0.999, indicative of high coverage similar to the rarefaction curves (Figure S1). The dominant flora at the phylum level were Firmicutes followed by Proteobacteria, with Firmicutes accounting for 77.6~90.6% and Proteobacteria representing 4.2~20.9%. The remaining flora consisted of Bacteroidetes, Actinobacteria, Cyanobacteria, and others. *C. perfringens* infection increased the relative proportion of Firmicutes by 12.0% and decreased the relative proportion Proteobacteria by 72.7%. On the contrary, the proportion of Bacteroidota largely increased from 0.3% to 6.6%. Interestingly, although feeding Xyn appeared not to have an obvious effect on the composition of Bacteroidota, Xyn+Am and Xyn+Ap decreased Bacteroidota from 6.6% to 3.8% and 3.3%, respectively (Figure 5B and Table S5).

At the genus level, challenging with *C. perfringens* led to a significant reduction (*p* < 0.05) in the relative abundance of *Escherichia-Shigella* and an increase in *Faecalibacterium* (Figure 5C). The use of Xyn and Xyn+Am reverted the trend of change for *Faecalibacterium* (Figure 5D). A similar mode of change was also noticed for *Elsenbergiella*, whose abundance was 2.7%, 5.5%, 2.8%, 2.8%, and 4.7%, in the control, Cp, Xyn, Xyn+Am, and Xyn+Ap groups, respectively. In addition, *C. perfringens* dramatically increased the abundance of *Allistipes* from 0.3% to 6.6%, which was decreased to 3.8% and 3.3% in the groups of Xyn+Am and Xyn+Ap, respectively. *Lactobacillus* showed a distinct trend in all treatment groups. After *C. perfringens* challenge, its abundance increased by 64.7%, and after enzyme addition, its abundance increased from 2.8% to 3.4%, 5.0%, and 3.5%, respectively (Figure 5E and Table S6). Compared to the normal group, the abundance of *Butyricicoccus* decreased in the Cp group and increased significantly in the Xyn and Xyn+Am groups, while the percentage of the Xyn+Ap group was almost equal to the level of the control group (Figure 5F).

## 4. Discussion

Broiler chickens, particularly those under stress conditions such as sudden changes of diets, are vulnerable to *C. perfringens* infection [35]. *C. perfringens* can produce various toxins, thereby inducing NE in chickens and causing significant intestinal damage [36]. In this study, we aimed to investigate the impact of feeding the *Cb*Xyn10C xylanase, either alone or in combination with an amylase or a protease, on protecting chickens from the intestinal lesions incurred by *C. perfringens* infection. The rationale was that *Cb*Xyn10C releases xylo-oligosaccharides from the feeds, which promotes the proliferation of gut resident probiotic bacteria [21]. The metabolites produced by the probiotics will in turn inhibit the proliferation of pathogenic bacteria such as *C. perfringens*, leading to the maintenance of intestinal homeostasis [11]. Proteases can degrade the biofilms of *Staphylococcus aureus* and *Staphylococcus epidermidis* [37,38]. Herein, we discovered that they could also degrade the biofilm of *C. perfringens*, thus assisting in further alleviating the intestinal lesions incurred due to infection with this pathogen.

Among the three investigated xylanases, *Cb*Xyn10C was superior in stimulating faster growth of probiotic bacteria. Hence, it was selected for the challenge experiment. As the gut intestine is a highly competitive niche for both probiotic and pathogenic bacteria [39], *Cb*Xyn10C was hypothesized to have a larger chance than the two other xylanases to assist probiotics in establishing growth competence, thereby limiting the propagation of *C. perfringens*. One direct effect of probiotic bacteria proliferation is to inhibit the growth of pathogenic bacteria [40]. This is because many of the excretes (such as acid and antimicrobial peptides) from the probiotic bacteria can have a detrimental effect on the viability or the growth of the pathogenic bacteria [18,41]. Although the other two xylanases were not studied for the challenge experiment, as expected, alleviation of *C. perfringens* infection in chickens by *Cb*Xyn10C was indeed observed. In comparison to the other two enzymes, when the same amounts of enzymes were used to treat feeds, *Cb*Xyn10C appears to have released more reducing sugars than the other enzymes. The degradation products were different for the three xylanases, which could also contribute to the profiles of stimulated gut bacteria. Such selection of xylo-oligosaccharides has been documented for the gut bacteria *lactobacilli* [42] and *Roseburia* [43]. Xylanases have been frequently investigated in poultry and livestock animals [18,20,44,45]. However, most studies have focused only on their benefits to the nutritional value. Therefore, the current study suggests that the mode of action for stimulating growth of gut bacteria is also important for maximizing the function of the xylanases.

The enzymes used in this study did not improve growth performance of the chickens. Similar phenomena have also been observed by other researchers for xylanase [20], protease [46], and amylase [47].

There have been reports documenting the feeding of xylanases to chickens challenged with *C. perfringens*. However, although promising results have been recorded for brush border enzymes and morphological traits of the small intestine, no definitive improvement in intestine lesions was reported [10,48]. One previous investigation combined a xylanase with debranching enzymes, such as an arabinofuranosidase and a feruloyl esterase, to promote the growth performance of broiler chickens [49,50]; however, whether the enzyme alone or in cocktail could help to defend against infection with *C. perfringens* remains unknown. Therefore, for the first time, we demonstrated proof-of-concept that feeding a xylanase, particularly in combination with a protease, could significantly mitigate the detrimental effect of *C. perfringens* on the intestinal barrier. One may notice that the damages were not completely diminished, which is similar to the results with other antibiotics-replacing agents, such as probiotics and plant extracts, in *C. perfringens* challenge experiments [6,18,51,52,53]. Whether the defending ability of chickens against *C. perfringens* can be further improved by increasing the amounts of enzymes remains to be elucidated. The biofilm of *C. perfringens* is not uniform, but is instead composed of multiple components such as extracellular DNA, proteins, and polysaccharides [27]. Therefore, another possibility is that the alkaline protease is not powerful enough to depolymerize the biofilm. Still another possibility is that combinatorial manipulation of the gut microbiota and biofilm is theoretically inadequate. In that case, other agents, either enzymes or other compounds, acting from complementary aspects can be included for maximal eradication of this pathogen.

In the current study, we used seven *Lactobacillus*, three *Enterococcus*, and two *Pediococcus* strains as exemplary gut probiotic bacteria. These genera are well-documented in their ability to produce bioactive compounds that are inhibitory to gut pathogens [52,54,55,56]. Analysis of the microbiome indicated that feeding xylanases did increase the abundance of *Lactobacillus*. Nevertheless, this was complicated by the fact that challenging chickens with *C. perfringens* also led to an increase in *Lactobacillus* (Figure 5E). However, this phenomenon has been observed before by other researchers. It was possible that certain species of lactobacilli were more resistant to *C. perfringens* infection than the others [43,57]. A further analysis of the gut microbiome at the species level indicated that the Cp group and the three enzyme groups have the same trend of change. Therefore, despite the increase in the *Lactobacillus* genus abundance, feeding xylanases and the other two enzymes changed the composition of *Lactobacillus* species, which may have different resistance to and inhibitory effects on *C. perfringens* (Figure S2). The lactic acid produced by *Lactobacillus* spp. can be fermented by *Butyricicoccus* to produce butyric acid, which can provide energy for and maintain the integrity of intestinal epithelial cells. The interaction between lactic acid-producing bacteria and lactic acid-utilizing bacteria could contribute to maintaining the homeostasis of gut microbiota. However, the interaction between gut bacteria is complex, and pathogens could evolve higher resistance to gut probiotics. Therefore, when other pathogens are to be tested using a similar strategy, more gut probiotics and commensals such as *Bifidobacteria* and *Bacteroides* could be involved.

Although feeding *Cb*Xyn10C and its binary mixtures did not significantly improve the intestinal morphology in the broiler chickens challenged with *C. perfringens*, all enzymes did enhance the expression of key genes in maintaining the gut barrier. Moreover, as expected, supplementing *Cb*Xyn10C with the alkaline protease displayed the minimum intestinal lesion score and maximal expression of the key genes involved in maintaining the intestinal barrier integrity. Many of these genes were even expressed to a higher level than that in the control group. The superiority of Xyn+Ap to Xyn in stimulating expression of these genes could be perceived by the higher transcript levels of ZO-1 and occludin in the duodenum and claudin-1 and occludin in the ileum. It was hypothesized that this was likely through degradation of the biofilm generated by *C. perfringens*. In addition, the protease can likely degrade the hydrolase virulence factors that are secreted by *C. perfringens* upon its infection of the chickens. Degradation leads to the loss of crucial virulence factors, consequently slowing down the disease’s progression. When compared to Xyn, supplementing *Cb*Xyn10C with the amylase showed a slight improvement regarding the indicators but no large effect on alleviating NE. It is worth noting that serious diarrhea was observed in the Xyn+Am group (Figure 3D). It has been reported that increasing the amylase in the digestive tract can disrupt intestinal osmolality and cause diarrhea in animals [58]. Diarrhea can shorten the retention time of chyme, thereby reducing the opportunity for pathogenic bacteria to colonize in the gut. Therefore, amylase added to the complexity of the alleviation of gut lesions.

The invasion of a pathogenic bacterium in the gut can trigger a cascade of events, leading to increased serum levels of IL-6, TNF-*α*, and IL-1*β* [59]. This has also been observed in our study, reflecting an activated systemic immune response. Unexpectedly, the concentration of TNF-*α* was significantly elevated in two enzyme groups (Xyn and Xyn+Ap), although no change was recorded for chickens challenged with *C. perfringens*. Interestingly, some xylanases are reported to be able to downregulate TNF-*α* expression [22,60]. Thus, the observed difference in modulating TNF-*α* expression might be ascribed to, at least in part, the different xylanases used in this study. It should be noted that, although TNF-*α* is well-known for its pro-inflammatory role, accumulating evidence unequivocally indicates that this cytokine has also an anti-inflammatory function in the intestinal epithelium, which can be fulfilled by inducing local glucocorticoid synthesis [61]. Therefore, the mechanisms underlying increased expression of TNF-*α* upon feeding of *Cb*Xyn10C and its binary mixtures is thus intriguing and deserves to be elucidated in the future.

## 5. Conclusions

In summary, the *Cb*Xyn10C xylanase and a protease were selected to control necrotic enteritis in chickens due to their ability to inhibit growth and biofilm formation of *C. perfringens*, respectively. Oral administration of these two enzymes alleviated the intestinal lesions caused by *C. perfringens* infection. In addition, the two enzymes influenced the abundance of *Lactobacillus* and *Butyricicoccus* bacteria in the intestine. Therefore, employing enzymes with the ability to modulate the gut microbiota appears to be effective in combating NE in chickens, which may further be improved upon and extended to other intestinal infectious diseases in animals.

## Figures and Tables

**Table 1:** Composition and nutrient levels of the diets of broiler chickens used in this study.

**Item, %**	**Content**
Wheat ^1^	64.22
Soybean meal	28.60
Soybean oil	2.10
CaHPO_4_	1.70
*DL*-Methionine	0.17
*L*-Lysine	0.18
Threonine	0.13
Limestone	1.10
NaCl	0.30
Premix ^2^	1.00
TiO_2_	0.50
Total	100.00
**Nutrient levels (%)** ^3^	**Content**
ME/(kcal/kg)	2900.00
CP	21.99
Ca	1.00
TP	0.44
Methionine	0.50
Lysine	1.13
Threonine	0.89

^1^ By containing wheat, the diet was rich in non-starch polysaccharides (NSPs) and the risk of NE might be increased. ^2^ The premix provided the following per kg of diet: VA 10,000 IU, VD_3_ 2000 IU, VE 20 IU, VB_1_ 2.0 mg, VK_3_ 2.5 mg, VB_2_ 4.0 mg, VB_6_ 5.0 mg, VB_12_ 0.02 mg, D-pantothenic acid 11.0 mg, nicotinic acid 35 mg, folic acid 0.5 mg, biotin 0.12 mg, Fe (as ferrous sulfate) 80 mg, Cu (as copper sulfate) 8 mg, Zn (as zinc sulfate) 78 mg, Mn (as manganese sulfate) 100 mg, I (as potassium iodide) 0.34 mg, Se (as sodium selenite) 0.15 mg. CP: crude protein. TP: total phosphorus. ^3^ The quantification of all nutrient levels of the diets was performed in our laboratory after the feed was prepared.

**Table 2:** Effect of supplementing with *Cb*Xyn10C and its binary mixtures on the growth performance of broiler chickens when challenged with *C. perfringens*.

Treatments	BW- 14 Day,	BW- 20 Day,	14–20 Day			1–20 Day		
	g	g	ADG, g/d	ADFI, g/d	FCR	ADG, g/d	ADFI, g/d	FCR
Control	387.56 ^ab^	818.04 ^ab^	62.82 ^b^	94.97 ^c^	1.46	35.20	51.42	1.42
Cp	378.92 ^ab^	787.27 ^a^	58.33 ^a^	89.91 ^b^	1.54	33.92	50.10	1.48
Xyn	369.27 ^a^	790.67 ^a^	60.20 ^ab^	85.59 ^a^	1.43	34.08	48.79	1.43
Xyn+Am	384.45 ^ab^	826.69 ^b^	63.18 ^b^	90.06 ^b^	1.43	35.72	49.96	1.40
Xyn+Ap	407.91 ^b^	831.43 ^b^	60.50 ^ab^	89.80 ^b^	1.47	35.96	49.77	1.38
SEM	4.382	12.169	1.217	1.046	0.023	0.554	0.501	0.014
*p* value	0.038	0.017	0.018	0.003	0.494	0.133	0.082	0.262

^a–c^ Mean values (*n* = 6) within a column with different letters indicate the existence of significant difference (*p* < 0.05). The same letters on one column indicate that there is no statistical difference. control: no enzyme and no *C. perfringens* challenge; Cp: no enzyme, *C. perfringens* challenge; Xyn, Xyn+Am, and Xyn+Ap: basic diet supplemented with *Cb*Xyn10C, *Cb*Xyn10C+amylase, *Cb*Xyn10C+alkaline protease, all with *C. perfringens* challenge.

**Table 3:** Effect of being fed enzymes on the expression of intestinal barrier factors in broilers challenged with *C. perfringens*.

Intestinal Segment	Gene	Control	Cp	Xyn	Xyn+Am	Xyn+Ap	SEM	*p* Value
Duodenum	ZO-1	1.00 ^a^	0.76 ^a^	1.26 ^a^	1.00 ^a^	2.38 ^b^	0.112	0.000
	claudin-1	1.00 ^ab^	0.72 ^a^	1.03 ^ab^	1.30 ^b^	1.52 ^b^	0.079	0.001
	occludin	1.04 ^a^	0.79 ^a^	1.50 ^b^	2.81 ^c^	2.85 ^c^	0.179	0.000
	MUC-2	1.01 ^a^	1.22 ^ab^	1.52 ^b^	1.26 ^ab^	1.71 ^b^	0.068	0.010
Jejunum	ZO-1	1.05 ^b^	0.60 ^a^	0.73 ^a^	0.69 ^a^	0.74 ^a^	0.039	0.000
	claudin-1	1.00 ^ab^	0.65 ^a^	1.27 ^b^	1.11 ^ab^	1.35 ^b^	0.067	0.003
	occludin	1.02 ^b^	0.40 ^a^	0.49 ^a^	0.67 ^a^	0.61 ^a^	0.046	0.000
	MUC-2	1.01 ^b^	0.64 ^a^	0.62 ^a^	0.57 ^a^	0.53 ^a^	0.040	0.000
Ileum	ZO-1	1.00 ^ab^	0.54 ^a^	1.72 ^c^	1.22 ^bc^	1.38 ^bc^	0.092	0.004
	claudin-1	1.09 ^b^	0.5 ^a^	0.69 ^ab^	0.73 ^ab^	1.76 ^c^	0.094	0.003
	occludin	1.01 ^a^	0.63 ^a^	1.20 ^a^	1.11 ^a^	2.21 ^b^	0.007	0.001
	MUC-2	0.97 ^b^	0.42 ^a^	0.51 ^a^	0.50 ^a^	0.63 ^a^	0.053	0.000

^a–c^ Mean values (*n* = 6) within a row with different letters indicate the existence of significant difference (*p* < 0.05). The same letters on one row indicate that there is no statistical difference. control: no enzyme and no *C. perfringens* challenge; Cp: no enzyme, *C. perfringens* challenge; Xyn, Xyn+Am, and Xyn+Ap: basic diet supplemented with *Cb*Xyn10C, *Cb*Xyn10C+amylase, *Cb*Xyn10C+alkaline protease, all with *C. perfringens* challenge.

**Table 4:** Effect of being fed enzymes on serum biochemical indices in broilers challenged with *C. perfringens*.

Treatment	DAO (ng/mL)	IgA (pg/mL)	IgG (μg/mL)	IgM (ng/mL)	IL-6 (pg/mL)	IL-10 (pg/mL)	IL-1*β* (pg/mL)	TNF *α* (pg/mL)
Control	0.99 ^a^	330.59	7.91	207.61	11.95 ^a^	18.24	11.19 ^a^	20.48 ^a^
Cp	1.86 ^b^	386.54	9.40	227.93	27.69 ^c^	16.51	22.65 ^b^	22.61 ^a^
Xyn	1.65 ^b^	335.51	8.67	202.57	23.07 ^b^	15.94	20.29 ^b^	30.51 ^b^
Xyn+Am	1.72 ^b^	356.61	8.13	205.35	24.11 ^b^	21.09	20.22 ^b^	26.85 ^ab^
Xyn+Ap	1.43 ^ab^	347.08	8.81	216.95	23.08 ^b^	19.30	24.49 ^b^	34.67 ^b^
SEM	0.095	11.330	0.240	5.216	1.141	0.700	1.169	1.332
*p* value	0.008	0.575	0.314	0.558	0.000	0.118	0.001	0.001

^a–c^ Mean values (*n* = 6) within a column with different letters indicate the existence of significant difference (*p* < 0.05). The same letters on one column indicate that there is no statistical difference. control: no enzyme and no *C. perfringens* challenge; Cp: no enzyme, *C. perfringens* challenge; Xyn, Xyn+Am, and Xyn+Ap: basic diet supplemented with *Cb*Xyn10C, *Cb*Xyn10C+amylase, *Cb*Xyn10C+alkaline protease, all with *C. perfringens* challenge.

## Data Availability

The original contributions presented in the study are included in the article/Supplementary Materials; further inquiries can be directed to the corresponding author.

## Supplementary Materials

The following supporting information can be downloaded at: https://www.mdpi.com/article/10.3390/ani15020123/s1. Table S1: Primers used for RT-qPCR in this study; Table S2: The selected enteric probiotics inhibited growth of *C. perfringens*; Table S3: Effect of feeding enzymes on the intestinal morphology in broilers challenged with *C. perfringens*; Table S4: The sample richness and diversity of the cecal microbiota; Table S5: Relative abundance of the dominant bacteria in the cecum at the phylum level; Table S6: Relative abundance of the dominant bacteria in the cecum at the genus level; Figure S1: Rarefaction curves of samples clustered at 95% sequences identity; Figure S2: Kruskal-Wallis H test on species level.
