# Supplementary material for: Alleviating Clostridium perfringens-Induced Intestinal Lesions in Chickens Using the Xylanase CbXyn10C and Its Binary Cocktail with a Protease
Source: Animals (Basel). 2025 Jan 7;15(2):123. doi: 10.3390/ani15020123 (PMC11758347; doi:10.3390/ani15020123)
Supplement: Supplementary file 1 [file animals-15-00123-s001.zip › animals-3305704-supplementary.pdf]

Supplemental Materials

Table S1. Primers used for RT-qPCR in this study

Primer	Sequence (5'-3')	Usage
Forward	GAGAAATTGTGCGTGACATCA	Analysis of β-actin
Reverse	CCTGAACCTCTCATTGCCA	Analysis of β-actin
Forward	CATACTCCTGGGTCTGGTTGGT	Analysis of claudin-1
Reverse	GACAGCCATCCGCATCTTCT	Analysis of claudin-1
Forward	ACGGCAGCACCTACCTCAA	Analysis of occludin
Reverse	GGGCGAAGAAGCAGATGAG	Analysis of occludin
Forward	AGCCCCTTGGTAATGTGTGG	Analysis of ZO-1
Reverse	CCAGGTTTTGGGGTCACAGT	Analysis of ZO-1
Forward	TTCATGATGCCTGCTCTTGTG	Analysis of MUC 2
Reverse	CCTGAGCCTTGGTACATTCTTG	Analysis of MUC 2

Table S2. The selected enteric probiotics inhibited growth of *C. perfringens*

Genus	Species	Inhibition halo diameter
Lactobacillus	*L. reuteri*	+++
	L. ingluviei	+++
	L. brevis	+
	L. crispatus	+
	L. mucosae	+++
	L. salivarius	+++
	L. johnsonii	+
Enterococcus	*E. hirae*	+++
	E. durans	++
	E. faecium	++
Pediococcus	*P. acidilactici*	+++
	P. pentosaceus	+++

+, Inhibition halo of <0.5 cm radius; ++, Inhibition halo of 0.5-1.0 cm radius; +++, Inhibition halo of >1.0 cm radius.

Table S3. Effect of feeding enzymes on the intestinal morphology in broilers challenged with *C. perfringens*

Treatment	Duodenum VH (μm)	Duodenum CD (μm)	Duodenum VH/CD	Jejunum VH (μm)	Jejunum CD (μm)	Jejunum VH/CD	Ileum VH (μm)	Ileum CD (μm)	Ileum VH/CD
Control	1350.45	185.88	7.56	758.66	156.85	5.01	384.37	118.57	3.32
Cp	1273.07	218.87	5.90	737.96	175.51	4.25	394.81	145.48	2.90
Xyn	1347.51	192.12	7.01	811.19	172.17	4.76	418.89	129.50	3.31
Xyn+Am	1391.92	227.01	6.30	775.73	183.52	4.46	414.44	133.90	3.13
Xyn+Ap	1491.32	206.88	7.07	814.60	174.63	4.77	414.00	131.63	3.26
SEM	25.221	7.139	0.247	20.089	6.444	0.169	7.166	6.313	0.119
P value	0.077	0.079	0.207	0.729	0.792	0.681	0.507	0.786	0.797

All results are expressed as mean (*n*=6). Control: no enzyme and no *C. perfringens* challenging; Cp: no enzyme, *C. perfringens* challenging; Xyn, Xyn+Am, and Xyn+Ap: basic diet supplemented with *Cb*Xyn10C, *Cb*Xyn10C+amylase, and *Cb*Xyn10C+alkaline protease, respectively, all with *C. perfringens* challenging.

Table S4. The sample richness and diversity of the cecal microbiota

Group	Shannon	Simpson	Ace	Chao 1	Coverage
Control	3.30	0.09	243.44 ^{ab}	243.42 ^{ab}	0.999
Cp	3.41	0.08	266.31 ^{bc}	265.56 ^{bc}	0.999
Xyn	3.39	0.07	246.53 ^{ab}	247.11 ^{ab}	0.999
Xyn+Am	3.52	0.06	240.13 ^{a}	240.36 ^{a}	0.999
Xyn+Ap	3.43	0.08	273.78 ^{c}	278.41 ^{c}	0.999
SEM	3.047	0.006	4.276	4.398	0.000
P value	0.700	0.619	0.027	0.013	0.096

$^{a\text{-}c}$ Mean values (*n*=6) within a column with different letters were significantly different (*P*<0.05). Control: no enzyme and no *C. perfringens* challenging; Cp: no enzyme, *C. perfringens* challenging; Xyn, Xyn+Am, and Xyn+Ap: basic diet supplemented with *Cb*Xyn10C, *Cb*Xyn10C+amylase, *Cb*Xyn10C+alkaline protease, respectively, all with *C. perfringens* challenging.

Table S5. Relative abundance of the dominant bacteria in the cecum at the phylum level

Phylum	Control-Mean (%)	Control-SEM (%)	Cp-Mean (%)	Cp - SEM (%)	Xyn-Mean (%)	Xyn - SEM (%)	Xyn+Am-Mean (%)	Xyn+Am-SEM (%)	Xyn+Ap-Mean (%)	Xyn+Ap-SEM (%)	*P* value
Firmicutes	77.59	4.91	86.91	3.06	75.7	11.33	86.8	1.61	90.64	3.07	0.431
Proteobacteria	20.9 [b]	4.23	5.70 [a]	1.88	16.9 [ab]	9.76	8.03 [a]	1.65	5.33 [a]	1.92	0.040
Bacteroidota	0.30 [a]	0.29	6.61 [b]	1.72	6.52 [ab]	3.04	3.84 [ab]	2.42	3.31 [ab]	1.71	0.048
Actinobacteriota	1.21	0.50	0.80	0.30	0.92	0.21	1.36	0.40	0.71	0.33	0.772
Cyanobacteria	0.00	0.00	0.00	0.00	0.10	0.06	0.00	0.00	0.00	0.00	0.253

[a, b] Mean values (n=6) within a row with different letters were significantly different (P<0.05). Control: no enzyme and no *C. perfringens* challenging; Cp: no enzyme, *C. perfringens* challenging; Xyn, Xyn+Am, and Xyn+Ap: basic diet supplemented with *Cb*Xyn10C, *Cb*Xyn10C+amylase, *Cb*Xyn10C+alkaline protease, respectively, all with *C. perfringens* challenging.

Table S6. Relative abundance of the dominant bacteria in the cecum at the genus level

Species name	Control-Mean (%)	Control-SEM (%)	Cp-Mean (%)	Cp-SEM (%)	Xyn-Mean (%)	Xyn-SEM (%)	Xyn+Am-Mean (%)	Xyn+Am-SEM (%)	Xyn+Ap-Mean (%)	Xyn+Ap-SEM (%)	*P*_value
Streptococcus	12.00	2.00	14.07	2.56	8.78	2.78	10.91	1.89	14.56	4.24	0.702
Escherichia-Shigella	20.91 [b]	4.20	5.67 [a]	1.89	16.91 [b]	9.83	8.04 [a]	1.74	5.31 [a]	3.13	0.043
Faecalibacterium	5.91	4.89	13.14	5.38	8.62	4.67	6.10	4.22	15.60	1.62	0.489
Ruminococcus_torques_group	8.03	1.63	9.62	2.42	7.45	2.20	10.95	2.74	6.62	1.51	0.716
unclassified_f__*Lachnospiraceae*	7.92	1.51	6.81	0.73	7.00	1.64	10.82	2.40	6.64	0.80	0.720
Alistipes	0.34 [a]	0.26	6.56 [b]	1.74	6.50 [ab]	3.02	3.78 [ab]	2.41	3.30 [ab]	1.11	0.045
norank_f__*Ruminococcaceae*	4.22	1.01	4.04	1.65	3.78	1.45	3.82	0.67	2.89	0.71	0.853
Eisenbergiella	2.66	0.90	5.52	3.44	2.82	1.12	2.80	0.89	4.67	0.62	0.467
Subdoligranulum	3.74	1.20	2.01	0.72	3.12	0.78	5.23	2.00	3.44	1.80	0.534
Lactobacillus	1.70 [a]	0.61	2.78 [b]	0.67	3.40 [b]	1.62	5.04 [c]	1.24	3.45 [b]	0.67	0.021
Erysipelatoclostridium	3.93 [b]	1.25	2.82 [ab]	0.54	2.21 [a]	0.93	3.28 [ab]	1.23	3.30 [ab]	0.89	0.035
unclassified_f__*Ruminococcaceae*	4.21	1.28	1.70	0.52	1.78	0.70	2.82	1.11	1.21	0.33	0.521
Butyricicoccus	1.62 [a]	0.73	1.51 [a]	0.35	3.02 [b]	1.10	3.21 [b]	1.30	1.34 [a]	0.20	0.036

[a-c] Mean values (n=6) within a row with different letters were significantly different (P<0.05). Control: no enzyme and no *C. perfringens* challenging; Cp: no enzyme, *C. perfringens* challenging; Xyn, Xyn+Am, and Xyn+Ap: basic diet supplemented with *Cb*Xyn10C, *Cb*Xyn10C+amylase, *Cb*Xyn10C+alkaline protease, respectively, all with *C. perfringens* challenging. Kruskal–Wallis rank sum test acted as the statistical significance test.

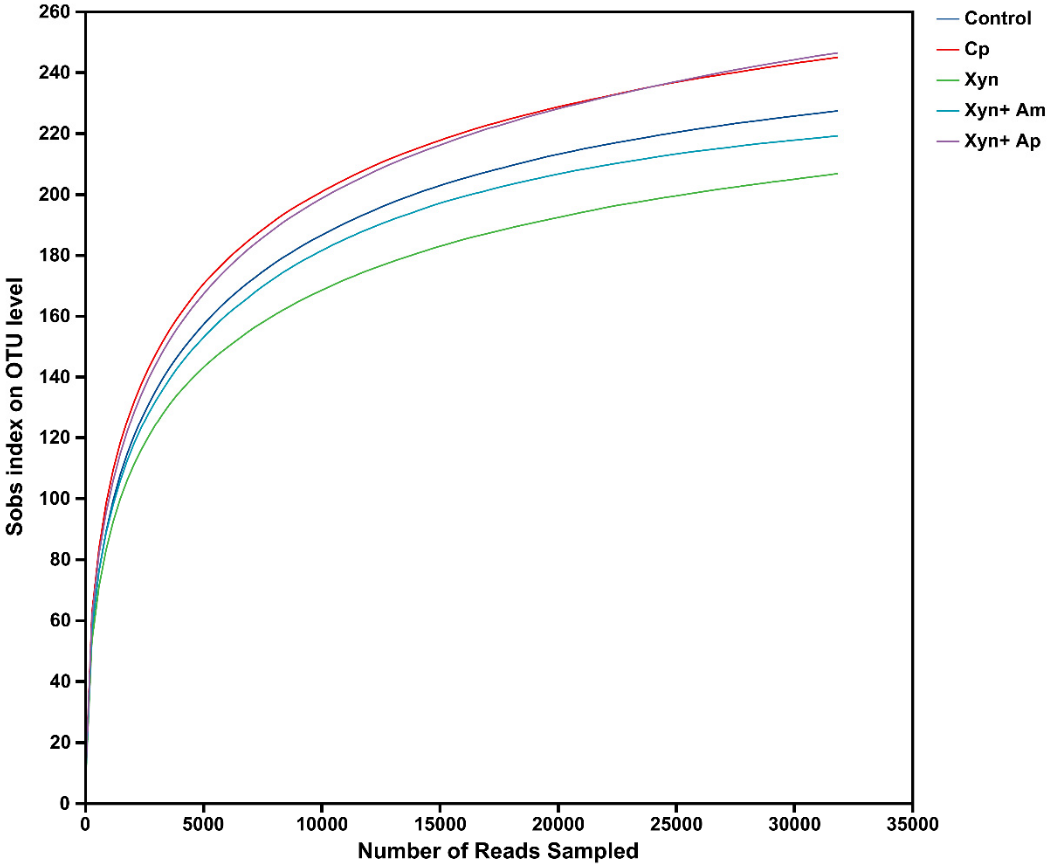

Figure S1. Rarefaction curves of samples clustered at 95% sequences identity.

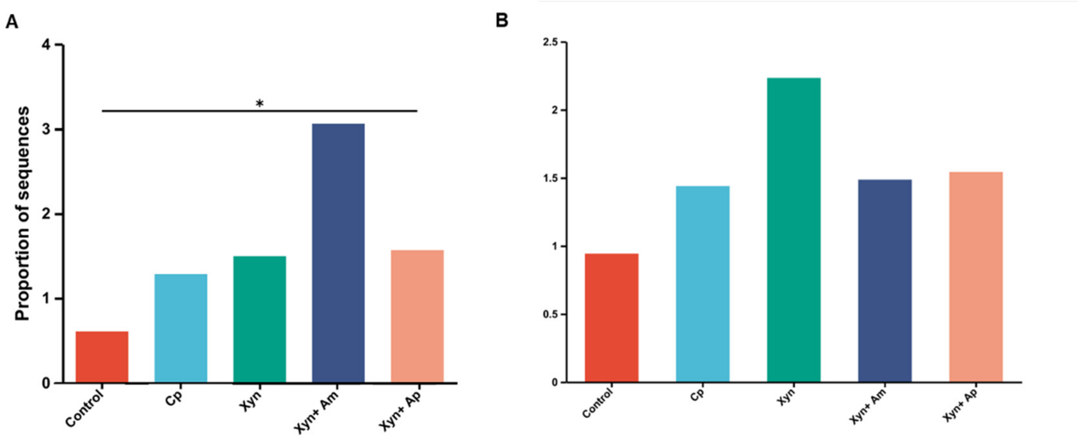

Figure S2. Kruskal-Wallis H test on species level. A: unclassified_g__*Lactobacillus*; B: *Lactobacillus salivarius*. * Indicates statistical difference (P<0.05).
